# Phenotype-Specific Heterogeneity in Acute Kidney Injury, Dialysis, and Mortality Among Hospitalized Patients with Chronic Kidney Disease: A National Retrospective Cross-Sectional Study

**DOI:** 10.3390/jcm15103593

**Published:** 2026-05-08

**Authors:** Brent Tai, Chijioke Okonkwo, Derek Snyder

**Affiliations:** Department of Internal Medicine, BayCare Health System, Clearwater, FL 33759, USA; chijioke.okonkwo@baycare.org (C.O.); derek.snyder@baycare.org (D.S.)

**Keywords:** chronic kidney disease, acute kidney disease, dialysis, in-hospital mortality, phenotyping, multimorbidity, National Inpatient Sample, health services research, hospital outcomes, administrative data

## Abstract

**Background:** Hospitalized patients with chronic kidney disease (CKD) are at high risk for acute kidney injury (AKI), dialysis, and mortality, yet CKD is often treated as a clinically homogeneous condition. Whether distinct cardiometabolic comorbidity patterns define meaningful inpatient CKD subgroups with differential outcome risks remains unclear. **Methods:** We conducted a retrospective cross-sectional study of adult hospitalizations for CKD using the 2022 Healthcare Cost and Utilization Project National Inpatient Sample. Hospitalizations were classified into five mutually exclusive CKD phenotypes using a rule-based framework based on diabetes mellitus, heart failure, hypertension, and vascular disease: isolated, hypertensive/vascular, metabolic, cardiorenal, and multimorbid cardiometabolic. Outcomes included AKI, dialysis during hospitalization, and in-hospital mortality. Survey-weighted multivariable logistic regression models were used to estimate adjusted odds ratios (aORs). Sensitivity analyses excluded end-stage kidney disease and dialysis dependence and restricted this study to non-transfer hospitalizations. The effect modification by age was assessed for dialysis. **Results:** Among 1,062,813 CKD hospitalizations, the unadjusted outcome rates varied substantially across phenotypes. After adjustment, cardiorenal CKD was associated with higher odds of acute kidney injury (aOR 1.16, 95% CI 1.12–1.19) and in-hospital mortality (aOR 1.54, 95% CI 1.50–1.58), whereas multimorbid cardiometabolic CKD demonstrated the strongest association with dialysis during hospitalization (aOR 2.34, 95% CI 2.25–2.43). Hypertensive/vascular CKD was not associated with a difference in mortality risk, while metabolic CKD was associated with a lower adjusted mortality rate compared to isolated CKD. Integrated analyses revealed distinct phenotype-specific risk profiles rather than a single severity gradient. Our findings were robust across the sensitivity analyses, and age significantly modified phenotype–dialysis associations. **Conclusions:** Hospitalized CKD populations exhibit marked phenotype-specific heterogeneity in AKI, dialysis, and mortality risk. A simple, clinically interpretable phenotype framework identifies distinct inpatient failure patterns and may inform future studies evaluating phenotype-specific risk stratification and management strategies.

## 1. Introduction

Chronic kidney disease (CKD) affects a substantial proportion of adults worldwide and is associated with high morbidity, mortality, and healthcare utilization, particularly during hospitalization [1,2,3]. Patients with CKD are disproportionately vulnerable to acute kidney injury (AKI), dialysis initiation, and in-hospital death, reflecting reduced renal reserves and the frequent coexistence of cardiometabolic comorbidities [4,5,6]. Despite this elevated risk, hospitalized CKD populations are often treated as clinically homogeneous, obscuring important differences in risk trajectories and limiting the ability to anticipate specific inpatient complications.

CKD rarely occurs in isolation and commonly coexists with diabetes mellitus, heart failure, hypertension, and vascular disease [7,8,9,10]. Each of these comorbid conditions contributes to distinct pathophysiologic stressors, including hemodynamic instability, metabolic dysregulation, and systemic inflammation, which may differentially influence inpatient outcomes [11,12]. Prior studies have largely examined these comorbidities individually or incorporated them into composite risk scores, approaches that may inadequately capture how combinations of cardiometabolic conditions interact to shape clinical risk during hospitalization.

Phenotype-based approaches offer a complementary framework for understanding disease heterogeneity by grouping patients according to clinically meaningful patterns rather than isolated variables. In chronic kidney disease (CKD), phenotyping is an area of active investigation, with an increasing use of data-driven approaches such as clustering and latent class analysis to identify clinically meaningful subgroups. These studies have consistently demonstrated that cardiometabolic comorbidities, particularly cardiovascular conditions, play a central role in defining the CKD phenotypic structure [13]. However, such approaches typically require high-dimensional clinical data, including laboratory values and longitudinal trajectories, which are not available in administrative datasets. As a result, their application to inpatient populations remains limited, and clinically interpretable approaches may offer a pragmatic alternative.

Moreover, existing inpatient studies of CKD have typically focused on single outcomes, such as AKI or mortality, without evaluating how different CKD subgroups may exhibit distinct modes of failure across multiple outcomes [14,15]. Understanding whether certain CKD phenotypes are preferentially associated with AKI, dialysis, or mortality could inform inpatient risk stratification, guide resource allocation, and support more targeted cardiorenal management strategies.

Accordingly, we sought to develop a clinically interpretable, rule-based CKD phenotype framework based on common cardiometabolic comorbidities and to evaluate its association with key in-hospital outcomes using a nationally representative cohort of hospitalized adults. Specifically, we examined unadjusted and adjusted associations between CKD phenotypes and AKI, dialysis during hospitalization, and in-hospital mortality and performed sensitivity and interaction analyses to assess robustness and effect modifications. Through this approach, we aimed to characterize phenotype-specific inpatient risk profiles and to demonstrate that hospitalized CKD populations exhibit meaningful heterogeneity that extends beyond traditional severity-based classifications.

## 2. Materials and Methods

### 2.1. Data Source and Study Design

We conducted a retrospective cross-sectional study using the 2022 Healthcare Cost and Utilization Project (HCUP) National Inpatient Sample (NIS). The NIS is a nationally representative database of inpatient hospitalizations in the United States, sampling approximately 20% of discharges from participating community hospitals and providing discharge-level weights to generate national estimates [16]. The unit of analysis in this study was the hospitalization rather than the individual patient. Because the NIS contains de-identified, publicly available data, this study was considered exempt from institutional review board review by the BayCare Health System (inquiry determination dated 22 October 2025).

### 2.2. Study Population and CKD Phenotype Classification

The study population included adult hospitalizations (aged ≥18 years) with a diagnosis of chronic kidney disease (CKD), identified using International Classification of Diseases, Tenth Revision, Clinical Modification (ICD-10-CM) diagnosis codes recorded during the index hospitalization. Hospitalizations with missing age information were excluded. As the NIS does not permit longitudinal patient tracking, individuals may have contributed more than one hospitalization during the study period, with each hospitalization treated as an independent observation.

CKD phenotypes were constructed using a rule-based clinical framework designed to reflect interpretable patterns of cardiometabolic comorbidity rather than latent disease severity. Four comorbid conditions were used to define phenotypes: diabetes mellitus, heart failure, hypertension, and vascular disease. Hospitalizations were categorized into five mutually exclusive phenotypes: isolated CKD (absence of all four comorbidities); hypertensive/vascular CKD (presence of hypertension and/or vascular disease without diabetes or heart failure); metabolic CKD (presence of diabetes mellitus without heart failure); cardiorenal CKD (presence of heart failure without diabetes); and multimorbid cardiometabolic CKD (presence of both diabetes mellitus and heart failure, with or without additional cardiometabolic comorbidities).

The selection of phenotype-defining comorbidities was guided by prior literature demonstrating the central role of cardiometabolic conditions, including diabetes mellitus, heart failure, hypertension, and vascular disease, in the CKD pathogenesis and progression. These conditions are consistently identified as dominant features in both clinical and data-driven CKD phenotyping studies. Given the limitations of the Nationwide Inpatient Sample, including the absence of laboratory data and longitudinal follow-up, a data-driven clustering approach was not feasible. Therefore, a rule-based framework was chosen to maximize clinical interpretability, reproducibility, and applicability within administrative data. This approach has not been formally validated and should be considered a pragmatic strategy for phenotype classification.

### 2.3. Outcomes and Covariates

The primary outcome was acute kidney injury (AKI) occurring during the index hospitalization. Secondary outcomes included dialysis during hospitalization and in-hospital mortality. Outcomes were ascertained using ICD-10-CM diagnosis codes and ICD-10-PCS procedure codes recorded during hospitalization. Dialysis was defined by the presence of inpatient dialysis procedure codes regardless of chronicity, an approach further evaluated in sensitivity analyses. All outcomes were assessed during the index hospitalization only. Dialysis during hospitalization was interpreted as an indicator of inpatient renal replacement therapy utilization rather than exclusively incident dialysis initiation and was therefore analyzed as a utilization-based outcome rather than a strict downstream consequence of acute kidney injury.

Patient-level covariates included age, sex, race/ethnicity, primary expected payer, and ZIP code-level median household income quartile. Hospital characteristics included teaching status, bed size, and geographic region. Comorbid conditions used to define CKD phenotypes were not included as covariates in adjusted models to avoid overadjustment and collinearity, as these variables constituted the exposure definition.

### 2.4. Statistical Analysis

All analyses accounted for the complex survey design of the NIS, incorporating discharge weights, hospital-level clustering, and stratification. Descriptive statistics were summarized as survey-weighted means with standard errors for continuous variables and weighted percentages for categorical variables. Unadjusted outcome rates across CKD phenotypes were summarized descriptively.

Survey-weighted multivariable logistic regression models were used to evaluate the association between CKD phenotype and each in-hospital outcome. Separate models were constructed for AKI, dialysis, and in-hospital mortality, with the CKD phenotype as the primary exposure and isolated CKD serving as the reference group. Models were adjusted for age, sex, race/ethnicity, primary payer, and ZIP code-level median household income quartile, and results were reported as adjusted odds ratios with 95% confidence intervals.

Sensitivity analyses were performed to assess the robustness of findings. Analyses were repeated after excluding hospitalizations with end-stage kidney disease or dialysis dependence and after restricting the cohort to non-transfer hospitalizations. Effect modification by age group (<65 vs. ≥65 years) was evaluated by including an interaction term between the CKD phenotype and age group for the dialysis outcome. All analyses were conducted using R (R Foundation for Statistical Computing, Vienna, Austria, ver. 4.5.2) [17], with survey-weighted analyses performed using the survey (ver 4.5) and srvyr packages (ver 1.3.0) and figures generated using ggplot2 (ver 4.0.1) and DiagrammeR (ver. 1.0.12).

## 3. Results

### 3.1. Study Cohort and CKD Phenotype Distribution

The final analytic cohort included 1,062,813 adult hospitalizations with chronic kidney disease (CKD). Using the predefined rule-based classification framework, hospitalizations were categorized into five mutually exclusive CKD phenotypes: isolated CKD (n = 236,447), hypertensive/vascular CKD (n = 39,074), metabolic CKD (n = 288,300), cardiorenal CKD (n = 203,556), and multimorbid cardiometabolic CKD (n = 295,436) (Table 1).

Table 1 summarizes the baseline demographic, clinical, and socioeconomic characteristics of hospitalized adults with chronic kidney disease, stratified by clinically defined CKD phenotypes. Values are survey-weighted means with standard errors or weighted percentages, as appropriate. Column headers report unweighted numbers of hospitalizations. Percentages may not sum to 100 due to rounding and missing data. Reported cardiometabolic comorbidities reflect phenotype-defining criteria rather than independent prevalence estimates. All estimates account for the complex survey design of the National Inpatient Sample.

Baseline demographic, clinical, and socioeconomic characteristics varied across CKD phenotypes (Table 1). Patients with cardiorenal CKD were older, with a mean age of 74.4 ± 0.03 years, compared with 69.5 ± 0.04 years among those with isolated CKD and 68.6 ± 0.03 years among those with metabolic CKD. The proportion of female patients was similar across phenotypes (approximately 44–48%). Racial compositions varied modestly, with Black patients accounting for approximately 14–22% of hospitalizations across phenotypes and Hispanic patients comprising the largest proportion in the metabolic CKD group (13.8%). Medicare was the predominant payer, representing 71–78% of hospitalizations depending on phenotype. The socioeconomic distribution by the ZIP code income quartile was relatively similar across groups.

**Figure 1:**
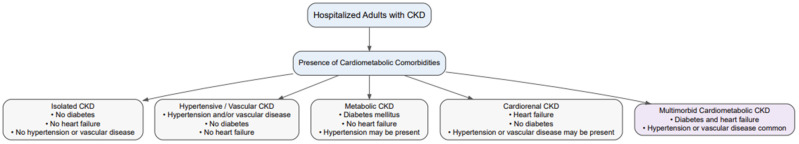
Conceptual framework for classification of chronic kidney disease phenotypes.

The conceptual framework used to define CKD phenotypes based on cardiometabolic comorbidity patterns is illustrated in Figure 1.

Figure 1 illustrates that hospitalized adults with chronic kidney disease (CKD) were categorized into five mutually exclusive phenotypes based on the presence or absence of key cardiometabolic comorbidities, including diabetes mellitus, heart failure, hypertension, and vascular disease. Phenotypes were defined using rule-based clinical criteria to reflect interpretable patterns of comorbidity rather than disease severity.

### 3.2. Unadjusted In-Hospital Outcomes Across CKD Phenotypes

**Figure 2:**
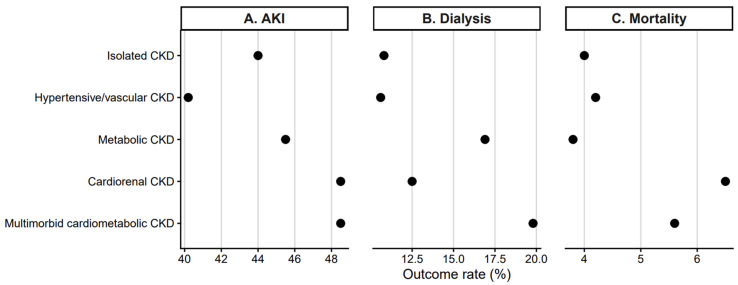
Unadjusted rates of acute kidney injury, dialysis, and in-hospital mortality across chronic kidney disease phenotypes.

Unadjusted in-hospital outcomes varied substantially across CKD phenotypes (Table 2; Figure 2). The rate of acute kidney injury (AKI) ranged from 40.2% in hypertensive/vascular CKD to 48.5% in cardiorenal CKD. Intermediate AKI rates were observed in metabolic CKD (45.5%), multimorbid cardiometabolic CKD (48.5%), and isolated CKD (44.0%).

Table 2 presents unadjusted in-hospital outcomes across clinically defined CKD phenotypes, including acute kidney injury, dialysis initiation, and in-hospital mortality. Values are shown as unweighted counts with survey-weighted percentages in parentheses. All estimates account for the complex survey design of the HCUP National Inpatient Sample. Acute kidney injury and dialysis were identified using ICD-10 diagnosis and procedure codes recorded during hospitalization.

Figure 2 illustrates unadjusted rates of acute kidney injury, dialysis, and in-hospital mortality varied across CKD phenotypes. Points represent survey-weighted percentages of each outcome within CKD phenotypes. The dashed vertical line indicates the reference value (OR = 1). Panel A displays acute kidney injury, Panel B displays dialysis during hospitalization, and Panel C displays in-hospital mortality.

Dialysis during hospitalization occurred most frequently among patients with multimorbid cardiometabolic CKD (19.8%), followed by metabolic CKD (16.9%), cardiorenal CKD (12.5%), isolated CKD (10.8%), and hypertensive/vascular CKD (10.6%).

In-hospital mortality was highest among patients with cardiorenal CKD (6.5%), followed by multimorbid cardiometabolic CKD (5.6%), hypertensive/vascular CKD (4.2%), and isolated CKD (4.0%). Metabolic CKD exhibited the lowest mortality rates (3.8%).

Taken together, these findings demonstrate substantial heterogeneity in the raw outcome burden across CKD phenotypes.

### 3.3. Adjusted Associations Between CKD Phenotypes and In-Hospital Outcomes

**Figure 3:**
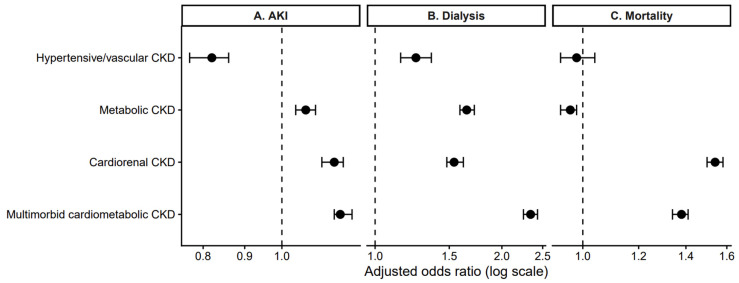
Adjusted associations between chronic kidney disease (CKD) phenotype and in-hospital outcomes.

After the adjustment for demographic and socioeconomic factors, the CKD phenotype remained independently associated with AKI, dialysis during hospitalization, and in-hospital mortality (Table 3; Figure 3).

Table 3 presents adjusted associations between clinically defined CKD phenotypes and in-hospital outcomes. Odds ratios (aORs) with 95% confidence intervals were estimated using survey-weighted multivariable logistic regression models adjusted for age, sex, race, primary payer, and ZIP code–level median household income quartile. Isolated CKD served as the reference phenotype.

Figure 3 illustrates the adjusted associations between chronic kidney disease phenotypes and in-hospital outcomes, demonstrating whether phenotype-specific differences persist after multivariable adjustment. Points represent adjusted odds ratios (aORs) with 95% confidence intervals from survey-weighted multivariable logistic regression models adjusted for age, sex, race, primary payer, and ZIP code-level median household income quartile. The dashed vertical line indicates the reference value (OR = 1). Panel A displays acute kidney injury, Panel B displays dialysis during hospitalization, and Panel C displays in-hospital mortality.

Compared with isolated CKD, cardiorenal CKD was associated with increased odds of acute kidney injury (adjusted odds ratio [aOR] 1.16, 95% CI 1.12–1.19) and substantially higher odds of in-hospital mortality (aOR 1.54, 95% CI 1.50–1.58). In contrast, hypertensive/vascular CKD was not associated with a difference in mortality risk (aOR 0.98, 95% CI 0.93–1.04), whereas metabolic CKD was associated with lower mortality compared with isolated CKD (aOR 0.96, 95% CI 0.93–0.98).

For dialysis during hospitalization, the strongest association was observed for multimorbid cardiometabolic CKD (aOR 2.34, 95% CI 2.25–2.43). Cardiorenal CKD (aOR 1.54, 95% CI 1.48–1.62) and metabolic CKD (aOR 1.65, 95% CI 1.59–1.72) were also associated with an increased dialysis risk, whereas hypertensive/vascular CKD demonstrated a more modest increase (aOR 1.25, 95% CI 1.15–1.36).

Notably, the dialysis risk did not parallel the AKI risk across phenotypes. While cardiorenal CKD demonstrated a consistently elevated risk across both AKI and mortality variables, multimorbid cardiometabolic CKD exhibited a disproportionately higher association with dialysis utilization despite only modestly increased odds of AKI. These findings suggest that dialysis during hospitalization may represent a distinct inpatient failure pathway rather than a direct extension of AKI severity.

### 3.4. Integrated Phenotype-Specific Risk Profiles

**Figure 4:**
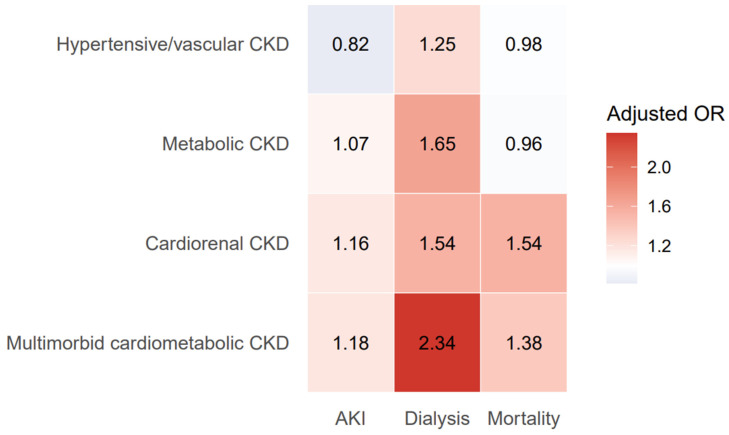
Heatmap of adjusted risk profiles across chronic kidney disease phenotypes and in-hospital outcomes.

When outcomes were considered jointly, CKD phenotypes demonstrated distinct and outcome-specific vulnerability patterns rather than a single continuum of disease severity (Figure 4).

Figure 4 synthesizes adjusted associations across outcomes to illustrate phenotype-specific risk profiles for acute kidney injury, dialysis, and in-hospital mortality. Colors represent the magnitude of adjusted odds ratios for acute kidney injury, dialysis during hospitalization, and in-hospital mortality across CKD phenotypes. Adjusted odds ratios were derived from survey-weighted multivariable logistic regression models, with isolated CKD serving as the reference phenotype. Warmer colors indicate higher relative risk, whereas cooler colors indicate lower relative risk.

Cardiorenal CKD was associated with increased odds of both AKI and mortality, reflecting a distinct pattern of risk across outcomes. In contrast, multimorbid cardiometabolic CKD demonstrated a dialysis-dominant profile, characterized by the strongest association with inpatient dialysis utilization. Hypertensive/vascular CKD consistently exhibited a lower or neutral relative risk across outcomes, whereas metabolic CKD exhibited a mixed pattern, with lower mortality but increased dialysis risk.

### 3.5. Sensitivity Analyses

Findings were robust across multiple sensitivity analyses. After excluding hospitalizations with end-stage kidney disease or dialysis dependence, phenotype-specific associations remained consistent (Supplementary Table S1). In this analysis, multimorbid cardiometabolic CKD demonstrated the highest risk of AKI (aOR 1.48, 95% CI 1.46–1.50) and remained strongly associated with dialysis (aOR 2.18, 95% CI 2.07–2.30) and in-hospital mortality (aOR 1.36, 95% CI 1.32–1.40). Cardiorenal CKD also exhibited strengthened associations across outcomes, including AKI (aOR 1.29, 95% CI 1.28–1.31), dialysis (aOR 1.56, 95% CI 1.47–1.66), and mortality (aOR 1.54, 95% CI 1.50–1.59).

When analyses were restricted to non-transfer hospitalizations, the overall findings remained largely unchanged (Supplementary Table S2). In this analysis, cardiorenal CKD remained associated with increased odds of AKI (aOR 1.13, 95% CI 1.11–1.15), indicating only modest attenuation compared with the primary analysis. Associations with dialysis and mortality were similarly preserved across phenotypes.

### 3.6. Effect Modification by Age

Age significantly modified the association between the CKD phenotype and dialysis during hospitalization (Supplementary Table S3). Among patients younger than 65 years, cardiorenal CKD was associated with an increased dialysis risk (aOR 1.48, 95% CI 1.44–1.52), with a similar magnitude of association observed among patients 65 years or older (aOR 1.50, 95% CI 1.46–1.54). In contrast, metabolic CKD demonstrated a stronger association with dialysis among older adults (aOR 1.59, 95% CI 1.55–1.63) compared with younger patients (aOR 1.35, 95% CI 1.31–1.38). Multimorbid cardiometabolic CKD remained strongly associated with dialysis in both age groups, with a more pronounced effect among older patients (aOR 2.34 vs. 1.73).

These findings indicate that the relationship between the cardiometabolic phenotype and renal replacement therapy utilization varies across age strata.

## 4. Discussion

In this nationwide analysis of hospitalized adults with chronic kidney disease (CKD), we identified clinically interpretable CKD phenotypes that were associated with distinct and outcome-specific patterns of acute kidney injury (AKI), dialysis, and in-hospital mortality. Rather than reflecting a single continuum of disease severity, these phenotypes exhibited divergent modes of inpatient failure, with differential vulnerability across renal and nonrenal outcomes. Together, these findings highlight the substantial heterogeneity within hospitalized CKD populations and underscore the value of phenotype-based frameworks for understanding inpatient risk.

The AKI risk varied substantially by phenotype, with both cardiorenal CKD and multimorbid cardiometabolic CKD demonstrating increased odds of AKI compared with isolated CKD after multivariable adjustments. This pattern was evident in unadjusted analyses and persisted after accounting for demographic and socioeconomic factors, suggesting that the cardiometabolic burden, particularly cardiac dysfunction, confers an increased vulnerability to acute renal decompensation in hospitalized CKD. In contrast, hypertensive/vascular CKD was associated with a lower AKI risk, whereas metabolic CKD demonstrated only a modest increase in risk. These phenotype-specific differences are visualized in the integrated risk heatmap (Figure 4), which highlights the elevated AKI risk across phenotypes characterized by greater cardiometabolic burdens.

Dialysis during hospitalization followed a markedly different pattern than AKI. Multimorbid cardiometabolic CKD exhibited the strongest association with dialysis, far exceeding that observed in other phenotypes. Importantly, the dialysis risk did not track directly with the AKI risk, reinforcing the concept that dialysis during hospitalization may represent a distinct pattern of association rather than a direct extension of AKI severity [18,19]. The dominance of the dialysis signal in multimorbid cardiometabolic CKD suggests that the combined metabolic and cardiac burden may lower thresholds for renal replacement therapy initiation or reflect advanced multisystem disease.

In-hospital mortality was highest among patients with cardiorenal CKD, with multimorbid cardiometabolic CKD also demonstrating an elevated mortality risk. These findings are consistent with the central role of cardiac dysfunction in driving adverse inpatient outcomes among patients with CKD [20,21]. Conversely, hypertensive/vascular CKD showed no significant difference in mortality, while metabolic CKD was associated with a slightly lower mortality risk despite increased dialysis utilization. These results suggest that cardiac involvement, rather than metabolic disease alone, is a key determinant of inpatient mortality risk in CKD.

A key contribution of this study is the demonstration that CKD phenotypes exhibit distinct failure patterns across outcomes, rather than differing only in their overall risk magnitude. Cardiorenal CKD is characterized by a combined AKI–mortality vulnerability, whereas multimorbid cardiometabolic CKD displays a dialysis-dominant risk profile. In contrast, hypertensive/vascular CKD consistently demonstrated a lower relative risk across outcomes, and metabolic CKD exhibited a mixed pattern with lower mortality but elevated dialysis risk. This integrated visualization emphasizes that CKD phenotypes do not lie along a single continuum of disease severity but instead fail in qualitatively different ways.

The observed phenotype–outcome associations were robust across multiple sensitivity analyses. Excluding hospitalizations with end-stage kidney disease or dialysis dependence yielded consistent results, with persistent associations across AKI, dialysis, and mortality among cardiorenal and multimorbid cardiometabolic CKD. Similarly, restricting analyses to non-transfer hospitalizations did not materially change the findings, with only modest attenuation in AKI associations for cardiorenal CKD. These findings support the stability of the phenotype framework and reduce concerns that results were driven by coding artifacts or referral bias.

We further identified age-dependent modifications of the association between the CKD phenotype and dialysis, with differential effects observed across age groups. While cardiorenal CKD demonstrated similar associations with dialysis across age strata, metabolic and multimorbid cardiometabolic CKD showed stronger associations among older patients. These findings highlight that age influences how cardiometabolic comorbidity patterns translate into renal replacement therapy utilization and suggest that phenotype-based risk stratification may require age-specific interpretation.

These findings have several potential clinical implications. First, a simple, rule-based CKD phenotype framework may inform future studies evaluating whether phenotype-specific risk patterns could guide clinical decision-making, including nephrology consultation and dialysis preparedness, beyond what can be inferred from a CKD diagnosis alone. Second, the recognition of dialysis-dominant versus AKI-dominant phenotypes may inform earlier nephrology consultation, dialysis preparedness, and multidisciplinary cardiorenal management. Finally, phenotype-based frameworks could support more nuanced inpatient risk stratification and resource allocation without a reliance on complex scoring systems.

Prior studies of CKD outcomes have often examined individual comorbidities or relied on composite risk prediction models, frequently treating CKD as a relatively homogeneous condition rather than a heterogeneous clinical syndrome [22]. Much of this literature has focused on outpatient populations and longitudinal risk stratification, particularly for predicting CKD progression or cardiovascular events [23]. However, these approaches may not fully capture the complex interplay of coexisting cardiometabolic conditions, and there is an increasing recognition of the need to more effectively characterize multimorbidity patterns in CKD. In contrast, this study integrates cardiometabolic comorbidity patterns into a unified, clinically interpretable phenotype framework and evaluates multiple inpatient outcomes simultaneously, thereby extending prior work and complementing existing outpatient risk models.

The CKD phenotype framework used in this study warrants further consideration. Phenotyping in CKD is an evolving area of research, with increasing applications of data-driven approaches, such as clustering and latent class analysis, to identify clinically meaningful subgroups. These approaches often require high-dimensional clinical data and longitudinal information, which are not available in administrative datasets such as the NIS. In this context, we employed a clinically informed, rule-based classification using major cardiometabolic comorbidities to provide a pragmatic and interpretable framework. While this approach enhances clinical applicability, it is not formally validated and should be considered hypothesis-generating. Future studies using data-driven methods may further refine these phenotypes and validate the observed associations.

This study has several limitations. First, its reliance on administrative data introduces the potential for the misclassification of CKD, comorbidities, and outcomes. Second, the NIS lacks laboratory data, including baseline creatinine values and measures of kidney function, as well as detailed clinical indicators of illness severity. As a result, residual confounding is likely. Differences observed between CKD phenotypes may therefore reflect variation in the underlying disease burden, severity of illness, or comorbidity complexity rather than distinct pathophysiologic subtypes. However, the consistency of associations across multiple outcomes and sensitivity analyses supports the robustness of the observed phenotype-specific risk patterns. Third, dialysis during hospitalization represents a utilization-based outcome and may include a heterogeneous population, including patients receiving maintenance dialysis, acute renal replacement therapy, or dialysis initiated based on varying clinical thresholds. As such, this measure should be interpreted cautiously and may reflect both clinical severity and differences in care processes rather than a uniform clinical endpoint. Fourth, the unit of analysis was the hospitalization rather than the individual patient, and repeat admissions could not be linked longitudinally. Residual confounding is possible despite the multivariable adjustment. Finally, the CKD phenotype classification was based on predefined combinations of comorbidities and has not been formally validated, and alternative data-driven approaches may identify different or more granular phenotypic subgroups.

In conclusion, hospitalized adults with CKD exhibit marked phenotype-specific heterogeneity in AKI, dialysis, and in-hospital mortality. Cardiorenal CKD is characterized by a heightened AKI and mortality risk, whereas multimorbid cardiometabolic CKD demonstrates a dialysis-dominant failure pattern. These findings suggest that CKD phenotypes provide a clinically meaningful framework for understanding inpatient risk and may inform future research on phenotype-specific risk stratification and management approaches in hospitalized CKD populations.

## Figures and Tables

**Table 1:** Baseline characteristics of hospitalized adults with chronic kidney disease by CKD phenotype.

Characteristic	Isolated CKD	Hypertensive/Vascular CKD	Metabolic CKD	Cardiorenal CKD	Multimorbid Cardiometabolic CKD
Unweighted count, n	236,447	39,074	288,300	203,556	295,436
**Demographics**					
Age, mean (SE), years	69.5 (0.04)	73.1 (0.07)	68.6 (0.03)	74.4 (0.03)	71.2 (0.02)
Female sex, %	48.6	44.2	45.8	46.2	45.8
**Race, %**					
White	67.4	72.4	56.0	68.5	59.0
Black	17.3	14.1	20.9	19.6	22.0
Hispanic	8.0	6.6	13.8	5.7	11.0
Asian or Pacific Islander	2.1	2.8	3.7	2.0	3.1
Native American	0.5	0.4	0.9	0.4	0.7
Other race	2.2	1.8	2.5	1.9	2.3
Missing	2.2	1.9	2.0	1.9	1.8
**Primary payer, %**					
Medicare	70.6	78.6	71.1	78.4	76.2
Medicaid	10.4	6.0	10.5	8.2	9.3
Private insurance	14.8	11.9	13.8	9.3	10.5
Self-pay	1.9	1.0	1.8	1.6	1.4
No charge	0.1	0.1	0.1	0.1	0.1
Other payer	2.5	2.4	2.6	2.3	2.6
**ZIP income quartile, %**					
Q1 (lowest)	27.8	25.8	32.3	29.3	32.9
Q2	25.5	25.2	25.9	25.4	26.3
Q3	24.1	25.1	23.1	24.0	23.1
Q4 (highest)	21.3	22.9	17.5	20.2	16.6
Missing	1.3	1.0	1.2	1.2	1.1
**Clinical comorbidities, %**					
Diabetes mellitus	0.0	0.0	100.0	0.0	100.0
Heart failure	0.0	0.0	0.0	100.0	100.0
Hypertension	0.0	17.4	2.7	0.5	0.4
Vascular disease	0.0	84.4	7.6	16.5	8.8

**Table 2:** In-hospital outcomes among hospitalized adults with chronic kidney disease.

Outcome	Isolated CKD (n = 236,447)	Hypertensive/Vascular CKD (n = 39,074)	Metabolic CKD (n = 288,300)	Cardiorenal CKD (n = 203,556)	Multimorbid Cardiometabolic CKD (n = 295,436)
Acute kidney injury	103,932 (44.0%)	15,701 (40.2%)	131,212 (45.5%)	98,816 (48.5%)	143,156 (48.5%)
Dialysis during hospitalization	25,628 (10.8%)	4125 (10.6%)	48,660 (16.9%)	25,480 (12.5%)	58,471 (19.8%)
In-hospital mortality	9498 (4.0%)	1640 (4.2%)	10,898 (3.8%)	13,293 (6.5%)	16,401 (5.6%)

**Table 3:** Adjusted associations between CKD phenotype and in-hospital outcomes.

CKD Phenotype	Acute Kidney Injury aOR (95% CI)	Dialysis During Hospitalization aOR (95% CI)	In-Hospital Mortality aOR (95% CI)
Isolated CKD	Reference	Reference	Reference
Hypertensive/vascular CKD	0.82 (0.77–0.86)	1.25 (1.15–1.36)	0.98 (0.93–1.04)
Metabolic CKD	1.07 (1.04–1.10)	1.65 (1.59–1.72)	0.96 (0.93–0.98)
Cardiorenal CKD	1.16 (1.12–1.19)	1.54 (1.48–1.62)	1.54 (1.50–1.58)
Multimorbid cardiometabolic CKD	1.18 (1.16–1.22)	2.34 (2.25–2.43)	1.38 (1.34–1.41)

## Data Availability

The data that support the findings of this study are available from the Agency for Healthcare Research and Quality, Department of Health and Human Services of the United States. However, restrictions apply to the availability of these data, which were used under license for the current study, and so are not publicly available. Data are, however, available from the author upon reasonable request and with permission of the Agency for Healthcare Research and Quality.

## Supplementary Materials

The following supporting information can be downloaded at https://www.mdpi.com/article/10.3390/jcm15103593/s1, Table S1: Sensitivity analysis excluding end-stage kidney disease and dialysis dependence; Table S2. Sensitivity analysis restricted to non-transfer hospitalizations; Table S3. Interaction between CKD phenotype and age group for dialysis during hospitalization.
