# Supplementary material for: Phenotype-Specific Heterogeneity in Acute Kidney Injury, Dialysis, and Mortality Among Hospitalized Patients with Chronic Kidney Disease: A National Retrospective Cross-Sectional Study
Source: J Clin Med. 2026 May 8;15(10):3593. doi: 10.3390/jcm15103593 (PMC13207225; doi:10.3390/jcm15103593)
Supplement: Supplementary file 1 [file jcm-15-03593-s001.zip › Supplementary Table 1.pdf]

Supplementary Table 1. Sensitivity analysis excluding end-stage kidney disease and dialysis dependence

CKD phenotype	Acute kidney injury aOR (95% CI)	Dialysis during hospitalization aOR (95% CI)	In-hospital mortality aOR (95% CI)
Isolated CKD	Reference	Reference	Reference
Hypertensive/vascular CKD	0.88 (0.86–0.90)	1.21 (1.08–1.35)	1.01 (0.96–1.08)
Metabolic CKD	1.22 (1.20–1.23)	1.32 (1.25–1.40)	0.96 (0.93–0.99)
Cardiorenal CKD	1.29 (1.28–1.31)	1.56 (1.47–1.66)	1.54 (1.50–1.59)
Multimorbid cardiometabolic CKD	1.48 (1.46–1.50)	2.18 (2.07–2.30)	1.36 (1.32–1.40)

Supplementary Table 1 presents adjusted associations between CKD phenotypes and in-hospital outcomes after excluding hospitalizations with end-stage kidney disease or dialysis dependence. Adjusted odds ratios (aORs) with 95% confidence intervals were estimated using survey-weighted multivariable logistic regression models after excluding hospitalizations with end-stage kidney disease (ICD-10-CM N18.6) or dialysis dependence (Z99.2). Models were adjusted for age, sex, race, primary payer, and ZIP code–level median household income quartile. Isolated CKD served as the reference phenotype. All analyses accounted for the complex survey design of the Healthcare Cost and Utilization Project National Inpatient Sample.
