# Supplementary material for: Phenotype-Specific Heterogeneity in Acute Kidney Injury, Dialysis, and Mortality Among Hospitalized Patients with Chronic Kidney Disease: A National Retrospective Cross-Sectional Study
Source: J Clin Med. 2026 May 8;15(10):3593. doi: 10.3390/jcm15103593 (PMC13207225; doi:10.3390/jcm15103593)
Supplement: Supplementary file 1 [file jcm-15-03593-s001.zip › Supplementary Table 2.pdf]

Supplementary Table 2. Sensitivity analysis restricted to non-transfer hospitalizations

CKD phenotype	Acute kidney injury aOR (95% CI)	Dialysis during hospitalization aOR (95% CI)	In-hospital mortality aOR (95% CI)
Isolated CKD	Reference	Reference	Reference
Hypertensive/vascular CKD	0.82 (0.80–0.84)	1.18 (1.13–1.24)	0.96 (0.90–1.02)
Metabolic CKD	1.10 (1.09–1.12)	1.54 (1.51–1.57)	1.00 (0.97–1.03)
Cardiorenal CKD	1.13 (1.11–1.15)	1.51 (1.47–1.55)	1.52 (1.47–1.57)
Multimorbid cardiometabolic CKD	1.18 (1.17–1.20)	2.13 (2.08–2.17)	1.40 (1.36–1.44)

Supplementary Table 2 presents adjusted associations between CKD phenotypes and in-hospital outcomes after restricting the cohort to non-transfer hospitalizations. Adjusted odds ratios (aORs) with 95% confidence intervals were estimated using survey-weighted multivariable logistic regression models restricted to non-transfer hospitalizations (excluding transfer-in and transfer-out admissions). Models were adjusted for age, sex, race, primary payer, and ZIP code–level median household income quartile. Isolated CKD served as the reference phenotype. All analyses accounted for the complex survey design of the Healthcare Cost and Utilization Project National Inpatient Sample.
