# Supplementary material for: Phenotype-Specific Heterogeneity in Acute Kidney Injury, Dialysis, and Mortality Among Hospitalized Patients with Chronic Kidney Disease: A National Retrospective Cross-Sectional Study
Source: J Clin Med. 2026 May 8;15(10):3593. doi: 10.3390/jcm15103593 (PMC13207225; doi:10.3390/jcm15103593)
Supplement: Supplementary file 1 [file jcm-15-03593-s001.zip › Supplementary Table 3.pdf]

Supplementary Table 3. Interaction between CKD phenotype and age group for dialysis during hospitalization

CKD phenotype	<65 years aOR (95% CI)	≥65 years aOR (95% CI)	P for interaction
Hypertensive/vascular CKD	1.30 (1.23–1.37)	1.11 (1.06–1.17)	<0.001
Metabolic CKD	1.35 (1.31–1.38)	1.59 (1.55–1.63)	<0.001
Cardiorenal CKD	1.48 (1.44–1.52)	1.50 (1.46–1.54)	<0.001
Multimorbid cardiometabolic CKD	1.73 (1.69–1.77)	2.34 (2.28–2.39)	0.014

Supplementary Table 3 presents age-stratified adjusted associations between clinically defined CKD phenotypes and dialysis during hospitalization, demonstrating effect modification by age group. Adjusted odds ratios (aORs) with 95% confidence intervals were estimated using survey-weighted multivariable logistic regression models including an interaction term between CKD phenotype and age group (<65 vs ≥65 years). Models were adjusted for sex, race, primary payer, and ZIP code–level median household income quartile. Isolated CKD served as the reference phenotype within each age stratum. All analyses accounted for the complex survey design of the Healthcare Cost and Utilization Project National Inpatient Sample.
